# Identification of the Sigma-2 Receptor: Distinct from the Progesterone Receptor Membrane Component 1 (PGRMC1)

**DOI:** 10.4172/2329-6488.1000e130

**Published:** 2016-04-25

**Authors:** Takato Hiranita

**Affiliations:** Division of Neurotoxicology, National Center for Toxicological Research (NCTR), U.S. Food and Drug Administration (FDA), USA

## Editorial

The sigma receptor (σR) subtypes, σ1 and σ2, have been mischaracterized [1,2]. A recent study suggested that the σ_2_R is the progesterone receptor membrane component 1 (PGRMC1) in rat livers. This finding was supported by the use of a novel photo affinity probe for σ_2_Rs, 5-[3-(4-[4azido2(4[6,7dimethoxy3,4dihydroisoquinolin 2(1H)yl]butylcarbamoyl)phenoxy]butyl)thioureido]-2-(6-hydroxy-3-oxo-3H-xanthen-9-l)benzoic acid (WC-21) [3]. Since that study, many have accepted that these two entities are the same. More recent studies have, however, indicated that this identification was mischaracterized [4,5]. This mischarecterization is significant for the establishment of σ_2_R pharmacology. Precise pharmacological characterization of the σ_2_R is important because it has been implicated with stimulant abuse [6,7].

σRs are unique intracellular chaperone proteins [8] initially thought to be opioid receptor subtypes [9]. They have been classified into two subtypes based on specific radioligand binding assays using [^3^H](+)-pentazocine for σ_1_Rs and [^3^H]1,3-di-o-tolylguanidine ([^3^H]DTG, in the presence of dextrallorphan to mask the σ_1_R) for σ_2_Rs in rat liver and kidney membranes [10]. Currently, the more selective σ_1_R ligand (+)-pentazocine has replaced dextrallorphan to mask the σ_1_R [7,11-14]. The σ_1_R has already been cloned as a 25-29 kDa chaperone protein composed of 223 amino acids [4,8,15]. It is widely distributed throughout the body [16-20]. Upon binding with agonists or under cellular stress, σ_1_Rs translocate from their primary endoplasmic reticulum (ER) location to different subcellular compartments where they can regulate ion channels and G-protein-coupled-receptor (GPCR) signaling [8,21-24]. *In vivo* functional studies on σ_1_Rs suggest that they play a substantial role in various cellular functions. Drugs acting at this receptor have been studied for their potential therapeutic effects in cancer, human immunodeficiency virus (HIV) infection, various psychiatric disorders, and substance abuse [1,25].

The σ_1_R is not a GPCR. Thus, it is challenging to determine what constitutes an agonist or an antagonist. For example, *in vitro* studies using NG-108 and Chinese Hamster Ovary (CHO) cells have demonstrated that the selective σ_1_R ligands PRE-084 and (+)pentazocine can dose-dependently cause the dissociation of σ_1_R from a binding immunoglobulin protein/78 kDa glucose-regulated protein (BiP/GRP-78), another ER chaperone [8,26]. Thus, they serve as agonists. In contrast, the σ_1_R ligands haloperidol and 4-methoxy-3-(2-phenylethoxy)-N,N-dipropylbenzeneethanamine (NE-100) alone do not affect the σ_1_R-BiP association but both completely inhibit the dissociation of σ_1_R from BiP caused by (+)pentazocine: they serve as antagonists [8,26]. In vivo, however, there is--as yet--no established functional assay for the σR subtypes. However, there is evidence showing a dose-dependent antagonism *in vivo* using the *in vitro* σ_1_R antagonists against the *in vitro* σ_1_R agonists using drug self-administration procedures [7,12,27,28]. Thus, it appears that the *in vitro* agonist-antagonist relationship will apply some *in vivo* responses.

The [^3^H] (+)-pentazocine-inaccessible σR, the σ_2_R, is an 18-21 kDa protein that has not been cloned yet [3,20,29-31]. However, a previous study using the radioligands [^3^H](+)-pentazocine, and [^3^H]DTG (in the presence of dextrallorphan) and a Flotillin-2 dotblotting technique in rat liver membranes found that σ_2_Rs are primarily localized in membrane lipid rafts whereas the σ_1_R localization appears in both raft and non-raft membrane domains [32]. The σ_1_R is dynamic and can translocate from its primary ER location to different subcellular compartments [24]. Previous mass spectrometry studies identified the σ2R-like proteins as being dimers consisting of H2A/H2B, the human nucleosomal proteins [33,34], which were defined using [^3^H]1-cyclohexyl-4-[3-(5-methoxy-1,2,3,4-tetra-hydronaphthalen-1-yl)propyl]piperazine ([^3^H]PB28) as a radioligand having a 19-fold higher affinity for the σ2 than for the σ1 receptors [35]. Abate et al. [34] showed that [^3^H]PB28 accumulation was up to five-fold higher in nuclear fractions than in cytosolic fractions in SK-N-SH and MCF7 cells. However, the dimer differs from the σ_2_R in membrane association [32]. Thus, the identity of σ_2_Rs as nucleosomal proteins does not appear to be viable.

Due to the lack of a known σ_2_R amino acid sequence, photoaffinity labeling remains the most viable approach for visualizing the receptor using sodium dodecyl sulfate (SDS) gels [29]. The basic principle is to covalently combine a photoactivatable σ_2_R-binding probe with the receptor such that the probe (radioactive- or fluorescent-labeled) remains with the protein even after denaturation with SDS [29]. Using a novel photoaffinity probe for σ_2_Rs, WC-21, a recent study identified the σ_2_R as the PGRMC1 in rat livers [3]. For example, the non-selective σ_1/2_R ligand DTG prevented the photolabeling of PGRMC1 (with WC-21) [3]. Further, an immunocytochemical study revealed that both PGRMC1 and (1R,3r,5S)-9-(10-[(7-Nitrobenzo[c] [1,2,5]oxadiazol-4-yl)amino]decyl)-9 azabicyclo[3.3.1]nonan-3-yl (2-methoxy-5-methylphenyl) carbamate (SW120), a fluorescent σ_2_R ligand, colocalize with molecular markers of the ER and mitochondria in HeLa cells [3]. As noted for the σ_1_R, studies utilizing various *in vitro* techniques indicated that σ_2_Rs are intracellular proteins. However, the affinity of DTG for the PGRMC1 was not reported in the study [3]. Nonetheless, it appears that the identification of the σ_2_R as the PGRMC1 [3] has been accepted widely. However, two recent studies [1,2] demonstrated a more viable data set against this identification as follows:

1. The molecular size of PGRMC1 (25 kDa) is approximately 7 kDa higher than that of the σ_2_R (∼ 18 kDa) [4].
2. Using specific photolabeling with [125I]-iodoazido-fenpropimorph ([125I]-IAF), the photolabeled σ_2_R band was not diminished in NSC34 cells devoid of or overexpressing the PGRMC1 [4]. Further, PGRMC1 knockout did not reduce [125I]-IAF photolabeling of the σ_2_R (18-21 kDa band) that was protectable by DTG and the highly σ_2_R-selective CM compounds [e.g. 1-(4-[6,7-dimethoxy-3,4-dihydroisoquinolin-2(1H)-yl]butyl)-3-methyl-1H-benzo[d]imidazol-2(3H)-one hydrochloride (CM 398)] [4]. The lack of influence of PGRMC1 knockout on the photolabeling of σ_2_R indicates a lack of a σ_2_R ligand-binding pocket formed by PGRMC1/σ_2_R complexes. The results also suggest that the σ_2_R is not a splice variant of the PGRMC1, thus, these two proteins are derived from different genes.
3. Alternatively, the PGRMC1 may be another DTG-binding protein that does not bind the photoprobe [125I]-IAF. If PGRMC1 is a high-affinity DTG binding site, elevation of PGRMC1 protein levels would result in an increase in maximal binding of [^3^H]DTG. However, neither the Bmax nor Kd values for [^3^H]DTG changed significantly in response to PGRMC1 overexpression, knockout or silencing in NSC34 cells [4] or human MCF7 adenocarcinoma cell lines [5] which are devoid of the σ_1_R [36].
4. Progesterone has been reported to be a high-affinity (Kd=35 nM) ligand for PGRMC1 (Table 1). However, the Ki value of progesterone for the σ_2_R [4] is approximately 406-fold higher than the Kd value for PGRMC1 in rat liver membranes (Table 1). Further, the Ki value of DTG for the PGRMC1 is 472,000 ± 420,000 nM (Table 1) using cold (+)-pentazocine to block the σ_1_R [4], which is approximately 15,000-fold higher than that for the σ_2_R [4] (Table 1). However, the Ki value of DTG for the PGRMC1 [4] was shown to be >1,000-fold lower than that obtained in a previous study [37] (Table 1). This discrepancy likely results from the lack of use of a selective cold blocker at the σ_1_R in the previous study [37] since DTG can also bind the σ_1_R with high affinity (Table 1). The binding profile of DTG for the PGRMC1 has been consistent with that for haloperidol, another non-selective σ1/2R ligand [4] (Table 1). Thus, the PGRMC1 is not a high-affinity DTG binding site, which also means that the PGRMC1 is not the σ_2_R.

Together, these new data [4,5] clearly suggest that the σ_2_R and PGRMC1 are two different molecular entities. Furthermore, the photo affinity probe containing a σ_2_R-directing moiety that led to the identification of PGRMC1 [3] as the σ_2_R (with WC-21), likely binds both σ_2_R and PGRMC1. The identification of the σ_2_R as distinct from the PGRMC1 [4,5] should have considerable impact especially in the cancer study field since the σ_2_R has been developed as a biomarker for various tumor cells [38]. Other studies have attempted to examine the correlation between the binding affinity of various σR ligands and their ability to produce effects both *in vitro* and *in vivo* through the σ_2_R [35,39]. However, the evidence for σ_2_R-mediated actions from these studies is not compelling because of the mixed use of σR agonist-like and antagonist-like ligands. Thus, the pharmacology and physiological role of σ_2_Rs remain undetermined due to unsuccessful efforts to clone the receptor and a lack of selective ligands. On the other hand, *in vitro* functional studies have demonstrated that the activation of the σ_2_R resulted in the synthesis and release of dopamine in the rat brain [6,7]. Thus, future studies that further explore σ_2_R pharmacology may result in a better understanding of the dopamine-mediated reinforcing mechanism associated with stimulant abuse and other dopamine-related diseases (e.g. Parkinson's disease and schizophrenia).

## Figures and Tables

**Table 1:** Inhibition (Ki values) by various compounds of specific binding to the σ1, σ2 receptors or PGRMC1. Values represent means ± SEM in nM. Values in parentheses are 95% confdence limits.

Compound	σ_1_R (26 kDa) [4]	σ_2_R (∼18 kDa) [4]	PGRMC1 (25 kDa) [4]
	[^3^H](+)-Pentazocine	[^3^H]DTG in the presence of (+)-pentazocine	[^3^H]Progesterone
(+)-Pentazocine	*3.38 (SEM=0.31) [5]	224 (95% confidence limits: 195-257) [13]	**63.9 [40]
DTG	57.4 (95% confidence limits: 49.3-66.7) [7]	*31.5 (SEM=3.3) [5]	472,000 (SEM=420,000) [4] 310 [37]
Haloperidol	2.91 (95% confidence limits: 2.69-3.14) [41]	31.5 (SEM=0.5) [4]	350,000 (SEM=19,000) [4]
Progesterone	1,540 (SEM=180) [42]	14,200 (SEM=4,900) [4]	*35 [43]

* Kd value

** IC_50_ values
